# Classification of short-term complications after transanal endorectal pullthrough for Hirschsprung’s disease using the Clavien–Dindo-grading system

**DOI:** 10.1007/s00383-019-04546-6

**Published:** 2019-08-14

**Authors:** Nils Hoff, Tomas Wester, Anna Löf Granström

**Affiliations:** 1 Department of Women’s and Children’s Health, Karolinska Institutet, Stockholm, Sweden; 2 Division of Pediatric Surgery, Astrid Lindgren Children’s Hospital, S3:02, Karolinska University Hospital, Solna, SE-17176 Stockholm, Sweden

**Keywords:** Hirschsprung disease, Transanal endorectal pullthrough, Clavien–Dindo, Complications

## Abstract

**Purpose:**

Hirschsprung’s disease (HSCR) is a developmental defect of the enteric nervous system. Transanal endorectal pullthrough (TERPT) is one of the surgical procedures for HSCR. Clavien–Dindo is an objective classification system, used worldwide, to describe postoperative complications. The aim of this study was to use Clavien–Dindo grading for short-term complication after TERPT.

**Methods:**

This was a cohort study including all 69 individuals, with biopsy-verified HSCR, managed with TERPT at our institution between 2006 and 2018. Data on the surgical procedure, as well as short-term complications, were retrieved from the medical records. The main outcome was postoperative complications graded according to Clavien–Dindo.

**Results:**

Fifteen (22%) of the 69 patients (51 males) had a short-term postoperative complication graded according to Clavien–Dindo. The complications were Grade I in ten patients, Grade II in four patients, and Grade IIIb in one patient. Individuals with a Clavien–Dindo complication had a significantly longer post-operative hospital stay [median 6 days (4–30) compared to 4 days (1–22), *p* = 0.035].

**Conclusions:**

It is important to describe postoperative complications in a structured way to make it possible to compare studies. Post-operative complications, according to Clavien–Dindo, occurred in 22% of the patients after TERPT.

## Introduction

Hirschsprung’s disease (HSCR) is a congenital malformation, characterized by the absence of ganglion cells in the distal hindgut. The absence of ganglion cells results in absent peristalsis in the affected bowel and the development of functional intestinal obstruction. HSCR is the most common congenital motility disorder in children, affecting 1 in 5000 live births [1]. HSCR is treated with rectosigmoid resection and coloanal anastomosis, using normally innervated colon for the anastomosis to the anus. In 1998, De la Torre described transanal endorectal pullthrough (TERPT). Modifications of this technique are frequently used today [2]. Short-term complications of TERPT have previously been described. Anastomotic leakage is the most serious early postoperative complications reported to occur in 0–1.5% of the patients [3, 4]. Anastomotic stricture, postoperative Hirschsprung-associated enterocolitis (HAEC), wound infections, bleeding, and perianal excoriations are other early post-operative complications [3, 4]. To be able to compare post-operative outcomes between different surgical centres, there is a need for an objective grading system of the early complications. Clavien–Dindo is a grading system used to objectively and reproducibly classify postoperative complications [5]. It consists of seven grades with increasing severity. Grade I being the most benign, stating that any deviation from the normal postoperative course without pharmacological treatment or surgical, endoscopic, and radiological interventions and Grade V is death (Table 1).

**Table 1:** Clavien–Dindo classification of surgical complications

Grade	Definition
Grade I	Any deviation from the normal postoperative course without the need for pharmacological treatment or surgical, endoscopic, and radiological interventions Allowed therapeutic regimens are: drugs as antiemetics, antipyretics, analgetics, diuretics, electrolytes, and physiotherapy. This grade also includes wound infections opened at the bedside
Grade II	Requiring pharmacological treatment with drugs other than such allowed for grade I complications. Blood transfusions and total parenteral nutrition are also included
Grade III	Requiring surgical, endoscopic or radiological intervention
IIIa	Intervention not under general anesthesia
IIIb	Intervention under general anesthesia
Grade IV	Life-threatening complication (including CNS complications)^a^ requiring IC/ICU management
IVa	Single organ dysfunction (including dialysis)
IVb	Multiorgan dysfunction
Grade V	Death of a patient
Suffix “d”	If the patient suffers from a complication at the time of discharge, the suffix “d” (for disability”) is added to the respective grade of complication. This label indicates the need for a follow-up to fully evaluate the complication

Presented as the description from: Classification of Surgical Complications, A New Proposal With Evaluation in a Cohort of 6336 Patients and Results of a Survey [5]

*CNS* central nervous system, *IC* intermediate care, *ICU* intensive care unit

^a^Brain hemorrhage, ischemic stroke, subarachnoidal bleeding, but excluding transient ischemic attacks

The aim of this study was to use Clavien–Dindo grading for short-term complication after TERPT and to investigate risk factors for developing a Clavien–Dindo short-term complication.

## Methods

This was a cohort study of patients who underwent surgery for HSCR between 2006 and 2018 at the Department of Pediatric Surgery, Karolinska University Hospital, Stockholm, Sweden. Patients with HSCR, confirmed with histology, were identified and included in the study. Data on HSCR and the surgical management were collected from the medical records. Exclusion criteria were patients who underwent other surgical procedures than TERPT including patient with total colonic aganglionosis (TCA). The follow-up period of the patients included the first 30 days after TERPT. Retrospective grading according to Clavien–Dindo was performed for each case as well as every type of complication that was registered (anastomotic leakage, stricture, postoperative HAEC, wound infections, and bleeding). Stricture was defined as a stricture treated with dilatation under general anesthesia.

### Statistical analysis

Categorical data were presented as frequencies or proportions and analyzed with two-tailed Fisher’s exact test. Numerous data were presented as mean and standard deviation if normally distributed, otherwise as medians and range. Continuous data were analyzed with *t* test for normally distributed data and Mann–Whitney *U* test for non-normally distributed data. Categorical variables were compared using Chi-square test. In all tests, *p* < 0.05 was considered statistically significant. Univariable logistic regression analyses were used to calculate the specific risk factors presented as odds ratio (OR) with the confidence interval (CI) set to 95%.

Statistical analyses were executed using IBM SPSS Statistics for Windows, version 25 (IBM Corp., Armonk, NY, USA) and MedCalc Statistical Software version 18.2.1 (MedCalc Software bvba, Ostend, Belgium; https://www.medcalc.org; 2016).

## Results

### Demographic data

During the time period from April 2006 until April 2018, 89 patients with HSCR managed at the Department of Pediatric Surgery of Karolinska University Hospital were identified. Twenty of these patients were excluded, six had undergone another surgical procedure, seven had TCA, three had tundergone TERPT at another institution, and four had HSCR, but had not yet undergone the surgical procedure. Sixty-nine (51 male) were finally included in the study. The median age at TERPT was 62 days (range 10–3355). Associated malformations were found in nine patients (13%). Ventricular septum defects were the most common associated malformation. Eight (11.6%) individuals had a syndrome, seven of whom had Down’s syndrome. Four individuals (5.8%) had HAEC before TERPT and ten patients (14.5%) had a stoma before TERPT. Demographic data are summarized in Table 2.

**Table 2:** Demographic data of the study population

	Total *n* = 69	Female *n* = 18	Male *n* = 51	Missing data	*p* value
Gender *n* (%)	69 (100)	18 (26.1)	51 (73.9)	0	
Associated malformations *n* (%)	9 (13)	1 (5.6)	8 (15.7)	0	0.428
Syndrome *n* (%)	8 (11.6)	0 (0)	8 (15.7)	0	0.101
Down’s syndrome *n* (%)	7 (10.1)	0 (0)	7 (13.7)	0	0.203
Heredity *n* (%)	9 (13)	6 (33.3)	3 (5.9)	0	**0.008**
Gestational age (weeks), median (range)	39 (30–42)	38 (30–42)	39 (31–42)	14 (5F/9M)	0.779
Birth weight, median, g (range)	3421 (1335–4500)	3200 (1335–4100)	3422 (1854–4500)	11 (5F/6M)	0.328
Initial symptoms *n* (%)					
Neonatal intestinal obstruction	58 (84.1)	13 (72.2)	45 (88.2)	0	0.140
HAEC	0 (0)	0 (0)	0 (0)	0	
Chronic constipation	11 (15.9)	5 (27.8)	6 (11.8)	0	
HAEC preop *n* (%)	4 (5.8)	1 (5.6)	3 (5.9)	0	1.000
Age at first rectal biopsy (days), median (range)	9.5 (2–3082)	13 (2–1067)	9 (3–3082)	5 (2F/3M)	0.378
Age at TERPT (days), median (range)	62 (10–3355)	66.5 (17–1158)	60 (10–3355)	0	0.477
Level of aganglionosis *n* (%)					
Rectum/rectosigmoid junction	61 (8.4)	15 (83.3)	46 (90.2)	0	0.421
Long segment (proximal to left flexure)	8 (10.7)	3 (15)	5 (9.1)	0	
Preoperative stoma *n* (%)	10 (14.5)	3 (16.7)	7 (13.7)	0	0.713

Significant result is in bold

Missing data shown under missing data, in total and how many females (F) and males (M) were missing, respectively

*HAEC* Hirschsprung-associated enterocolitis, *TERPT* transanal endorectal pullthrough

### Complication within 30 days of TERPT: Clavien–Dindo

Patients who had postoperative complications within 30 days of TERPT were graded using the Clavien–Dindo grading system. A total of 15 (22%) patients had a postoperative complication, ten of whom (14.4%) had a Grade I complication, four (5.8%) a Grade II, and 1 (1.4%) a Grade IIIb complication. All ten Grade I, complications were related to postoperative pain management. Three of the patients had an epidural catheter complication and seven of the patients received opiates not in compliance with a normal postoperative course. Four patients had a Grade II complication, one of whom required total parenteral nutrition, one had seizures and needed benzodiazepine, one had a superficial wound infection and required antibiotics, and one required blood transfusion post-operatively. One patient had a Grade IIIb complication caused by an abscess, which later needed a re-operation with an ileostomy. No death was reported.

Individuals with a Clavien–Dindo classified complication had a significantly longer postoperative hospital stay, median 6 days (range 4–30) compared to individuals without complications who had a median postoperative stay of 4 days (range 1–22), *p* = 0.035. One patient without a complication had a postoperative stay of 35 days due to Down’s syndrome with a severe congenital heart defect and heart failure and was excluded from the analysis of postoperative stay, as he was considered an outlier. The median postoperative hospital stay was 5 days (range 1–35). Eleven patients (15.9%) were readmitted within 30 days of TERPT and one patient was re-operated within 30 days of TERPT due to an abscess formation, as shown in Table 3.

**Table 3:** Complications within 30 days of TERPT, in total, female and male

	Total *N* = 69	Female *n* = 18	Male *n* = 51	*p* value
Postoperative complication < 30 days of TERPT *n* (%)	15 (21.7)	2 (11.1)	13 (25.5)	0.321
Clavien–Dindo classification *n* (%)				
Grade 0	54 (78.3)	16 (88.9)	38 (74.5)	0.599
Grade I	10 (14.5)	1 (5.6)	9 (17.6)	
Grade II	4 (5.8)	1 (5.6)	3 (5.9)	
Grade IIIb	1 (1.4)	0 (0)	1 (2)	
Anastomotic leakage *n* (%)	0 (0)	0 (0)	0 (0)	–
Stricture *n* (%)	12 (17.4)	1 (5.6)	11 (21.6)	0.163
Postoperative HAEC *n* (%)	23 (33.3)	3 (16.7)	20 (39.2)	0.144
Bleeding *n* (%)	1 (1.4)	0 (0)	1 (2)	1.0
Wound infection *n* (%)	2 (2.9)	0 (0)	2 (3.9)	1.0
Postoperative hospital (days), median (range)	5 (1–35)	4.5 (1–10)	5 (2–35)	0.593
Readmission within 30 days of TERPT *n* (%)	11 (15.9)	2 (11.1)	9 (17.6)	0.715
Reoperation within 30 days of TERPT (%)	1 (1.4)	0 (0)	1 (2)	1.0

*HAEC* Hirschsprung-associated enterocolitis, *TERPT* transanal endorectal pullthrough

### Risk factors for short-term complications

We found that there were no specific risk factors for developing a Clavien–Dindo short-term complication. All variables are shown in Table 4.

**Table 4:** Risk factors for developing a Clavien–Dindo classified short-term complication

	Univariate OR (95% CI)
Clavien–Dindo	
Male gender	0.37 (0.05–1.53)
Associated malformation	2.00 (0.38–8.82)
Syndrome	1.23 (0.17–6.11)
Heredity	0.41 (0.02–2.53)
Preoperative HAEC	1.21 (0.06–10.35)
Preoperative stoma	2.91 (0.65–12.06)
Age at TERPT days	2.97 (0.92–9.93)
Long segment	2.45 (0.45–11.49)

*HAEC* Hirschsprung-associated enterocolitis, *TERPT* transanal endorectal pullthrough

## Discussion

This is the first study that describes post-operative complications in patients with Hirschsprung’s disease graded according to Clavien–Dindo. A Clavien–Dindo complication was found in 15 (22%) of the patients. The purpose of this study was to highlight all possible adverse events after TERPT, such as inappropriate postoperative analgesia as well as prolonged time to oral feeding. To be able to compare results between different pediatric surgical centres, there is a need for an objective grading system also within pediatric surgery.

In this study, we used the Clavien–Dindo grading system, as described in 2004. The system has been widely used for evaluation of post-operative complications in general surgery [5]. The grading system has been sporadically used to evaluate outcomes in pediatric surgery, for example, to identify complications related to jejunal feeding and after repair for congenital duodenal obstructions [6, 7].

This was a single-centre study of individuals undergoing TERPT during a 12-year period. The grading was performed retrospectively after evaluating the first 30 postoperative days according to the grading system that Clavien–Dindo described. The advantages with this system are that all possible adverse events are included. The limitations are that the Clavien–Dindo grading was done retrospectively based on data from the medical records. This causes a risk for minor events, not noted in the medical records, to be unnoticed, while more serious events would not be missed. In this study, we found that having a Clavien–Dindo complication had a significant effect on post-operative hospital stay, indicating that also minor complications have a negative impact on the post-operative care. Regarding the complications usually described in the literature as anastomotic leakage, stricture, postoperative Hirschsprung-associated enterocolities (HAEC), wound infections, bleeding, and perianal excoriations, we believe that the Clavien–Dindo grading would identify all of them except for milder perianal excoriations.

To determine whether the short-term complications differed between the genders, we performed a gender analysis to see if they were prone to different types of complications, but no significant difference was found. This result may be a type II error due to the few females included in the study.

In this study, we could not identify any risk factors for developing a Clavien–Dindo complication, although there is an obvious risk for type II errors. We know from other studies that preoperative HAEC and length of aganglionosis are factors associated with post-operative complications, although this could not be confirmed in our study [8].

We chose to exclude individuals who had other surgical procedures than TERPT to be sure to compare the post-operative courses of one procedure. Individuals with TCA were excluded, since they also undergo a more extensive surgical procedure. Individuals who did not undergo TERPT at our clinic were excluded, since we could not properly evaluate them according to Clavien–Dindo.

We could not find any patient with an anastomotic leakage. Twelve patients (17.4%) had anastomotic strictures compared to 4–10% reported in the literature [3, 4]. This is partly a definition issue, and we have defined a stricture formation as a stricture in need for dilatation under general anaesthesia. Our study also correlates well with the 1.3% infection incidence reported by Teitelbaum et al. [9]. The patients in our study had a median hospital stay of 5 days, which is similar to a multicenter study by Elhalaby et al. showing a mean hospital stay of 4.8 days [10]. Twenty-two percent of the patients had a Clavien–Dindo complication, most of them low-grade. All low-grade complications (Grade I) were related to post-operative pain, which makes it necessary to address routines for postoperative analgesia. The Grade II was connected to non-surgical event in three of the patients, while one was caused by bleeding post-operatively. The only Grade IIIb complication was directly connected to the surgical procedure. This grading system reveals more about the post-operative course compared to only reporting post-operative in hospital stay and rates of leakage, stricture, and infections. We think that the usage of an objective classification scoring system for postoperative complication would make comparisons between different centres possible and make the results transparent.

## Conclusion

This is the first study to evaluate objectively post-operative complications in HSCR. Using the Clavien–Dindo grading showed that 22% of the patients had a post-operative complication, although most of them were Grade I. There is a need for further studies to evaluate objectively post-operative complications after pediatric surgical procedures.
